# Cardiac monoamine oxidases: at the heart of mitochondrial dysfunction

**DOI:** 10.1038/s41419-020-2251-4

**Published:** 2020-01-23

**Authors:** Jeanne Mialet-Perez, Angelo Parini

**Affiliations:** INSERM Institute of Metabolic and Cardiovascular Diseases (I2MC), Université de Toulouse, Toulouse, France

**Keywords:** Stress signalling, Mitochondria

Chronic postischemic remodeling is a major cause of mortality and morbidity worldwide. While ventricular remodeling involves complex mechanisms, energetic deficit, dysregulation of Ca^2+^ handling and oxidative stress are important hallmarks of the failing cardiomyocyte^1^. Mitochondria are at the centre of these processes as they are the main source of ATP and reactive oxygen species (ROS) and their function is critically controlled by Ca^2+^^2^.

Mitochondrial Ca^2+^ is necessary to match energy supply with the demand during excitation-contraction coupling through the regulation of the TCA cycle and the oxidative phosphorylation complexes. Thus, the ability of mitochondria to accumulate Ca^2+^ is fundamental for tissue homeostasis. The efficient flow of Ca^2+^ across the outer membrane requires mitochondria to be proximal to the endoplasmic reticulum (ER). These specific sites of association between ER and mitochondria delineate microdomains with high [Ca^2+^] that allow the transfer of Ca^2+^ through voltage-dependent anion channels (VDAC)^3^. Subsequently, uptake of Ca^2+^ across the inner membrane occurs through the recently identified mitochondrial Ca^2+^ uniporter (MCU). MCU alone is not sufficient for effective Ca^2+^ transfer but needs to be part of a macromolecular complex composed of a tetramer of MCU and several regulatory subunits (EMRE, MICU1, MICU2)^4^. Of particular importance, the oligomerization state of the MCU complex directly regulates mitoCa^2+^ uptake^5^.

Multiple evidences demonstrate that mitoCa^2+^ levels need to be fine-tuned in order to support efficient mitochondrial bioenergetics^2^. Abnormally high entry of Ca^2+^ seems to be detrimental for mitochondrial function. MitoCa^2+^ overload provoked by ER Ca^2+^ leak in mice mutated for the ryanodine receptors (RyR2) aggravated heart failure (HF) during myocardial infarction^6^. Also, deletion of NCLX, a channel that regulates mitoCa^2+^ efflux, caused spontaneous heart failure in mice^7^. While it is apparent that mitoCa^2+^ levels are dysregulated in HF, there is still a lack of comprehension of the mechanisms underscoring these effects. Furthermore, mitochondrial ROS are causally related to the progression of HF but the tight interplay between ROS and mitoCa^2+^ during ventricular remodeling remains incompletely understood.

One important source of ROS in the mitochondria is monoamine oxidase-A (MAO-A)^8^. MAO-A is an outer mitochondrial membrane enzyme that terminates noradrenaline signaling in the heart, but generates H_2_O_2_ as a byproduct during the degradation process. In situations of acute or chronic stress, we and others have shown that MAO-A was an important source of deleterious ROS, regulating cardiomyocyte senescence or death^9,10^. In a recent study, we focused on the role of MAO-A in ventricular remodeling during chronic ischemia, postulating that the chronic activation of sympathetic activity and the permanent release of catecholamines in this particular situation could fuel MAO-A activity^11^. By using gene-targeted approaches in mice (cardiomyocyte-specific overexpression or deletion), we demonstrated the deleterious role played by MAO-A in ventricular dysfunction during chronic ischemia^11^. Mechanistically, the excess of ROS generated by MAO-A led to an accumulation of 4-hydroxynonenal (4-HNE) inside the mitochondria. 4-HNE is a product of lipid peroxidation and a reactive aldehyde that is particularly deleterious since it is more long-lived than ROS and form adducts with proteins to modify their function and conformation. We first questioned how 4-HNE accumulated in response to MAO-A. In mice overexpressing MAO-A in the heart, we observed that mitochondria displayed decreased amounts of cardiolipins. Cardiolipins are phospholipids present only in the mitochondria and constituted of four linoleic moieties, the main precursor of 4-HNE. Following MAO-A activation, we observed an increase in mitochondrial concentrations of HODEs, the stable oxidation product of linoleic acid and intermediate to the synthesis of 4-HNE^11^. Thus, we demonstrated for the first time that activation of MAO-A and generation of H_2_O_2_ led to cardiolipin peroxidation and accumulation of mitochondrial 4-HNE. Next, we provided evidence that 4-HNE was a main contributor of MAO-A-associated ventricular dysfunction. By using an adeno-associated gene strategy with ALDH2, the main mitochondrial enzyme for degradation of 4-HNE, we conferred significant protection on 4-HNE accumulation, ventricular dysfunction and HF in MAO-A Tg mice^11^. Furthermore, Alda-1, a pharmacological activator of ALDH2, protected adult ventricular myocytes from 4-HNE accumulation, respiratory dysfunction and loss mitochondrial membrane potential induced by MAO-A. Finally, we searched for the specific mechanisms of action of 4-HNE in the heart. By using proteomic and biochemical analysis, we identified previously unrecognized targets for 4-HNE^11^. 4-HNE bound specifically to VDAC and MCU to regulate mitoCa^2+^ entry following MAO-A activation. MAO-A Tg mice exhibited higher levels of mitochondria-ER contact sites. In addition, binding of 4-HNE to the MCU led to the formation of MCU higher order oligomers, potentializing Ca^2+^ entry and leading to mitoCa^2+^ overload. These findings were recapitulated in chronic ischemia where inhibition of MAO-A prevented 4-HNE accumulation in the heart, higher order MCU oligomers formation and mitoCa^2+^ overload^11^.

Thus, we identified a cross-regulation between mitochondrial ROS and Ca^2+^ in chronic ventricular remodeling that is favored by MAO-A and impairs mitochondrial function. Simultaneous increases in mitoROS and Ca^2+^ have already been demonstrated in acute ischemia-reperfusion injury where they act in a synergistic way to regulate mitochondrial transition pore opening and cell death^12^. However, in chronic remodeling, such cross-regulation was not previously demonstrated. In addition, we identified a new mechanism by which ROS, through 4-HNE production, led to increased MCU activity and Ca^2+^uptake. Some other post-translational modifications on the MCU, such as phosphorylation by Pyk2, have also been shown to promote its oligomerization, enhancing channel activity^13^. This is also the case in a recent work by Dong et al. showing that oxidation and mutation of the Cys-97 of the MCU (a ROS-sensing residue) led to MCU higher-order oligomer formation finally resulting in persistent channel activity with higher [Ca^2+^]_m_ uptake rate^14^. As 4-HNE exhibits the strongest reactivity for Cys residues, it is possible that a similar mechanism operates following MAO-A activation. Finally, the downstream effects of mitoCa^2+^ overload on mitochondrial dysfunction and ATP depletion still lack an explanation at the moment. It is possible that excessive mitoCa^2+^, in turn, leads to further generation of ROS^2^. Also, as recently shown, mitoCa^2+^ could regulate mitochondrial morphology since overexpression of MCU in neurons led to mitochondrial fission and cell death^15^.

**Fig. 1:**
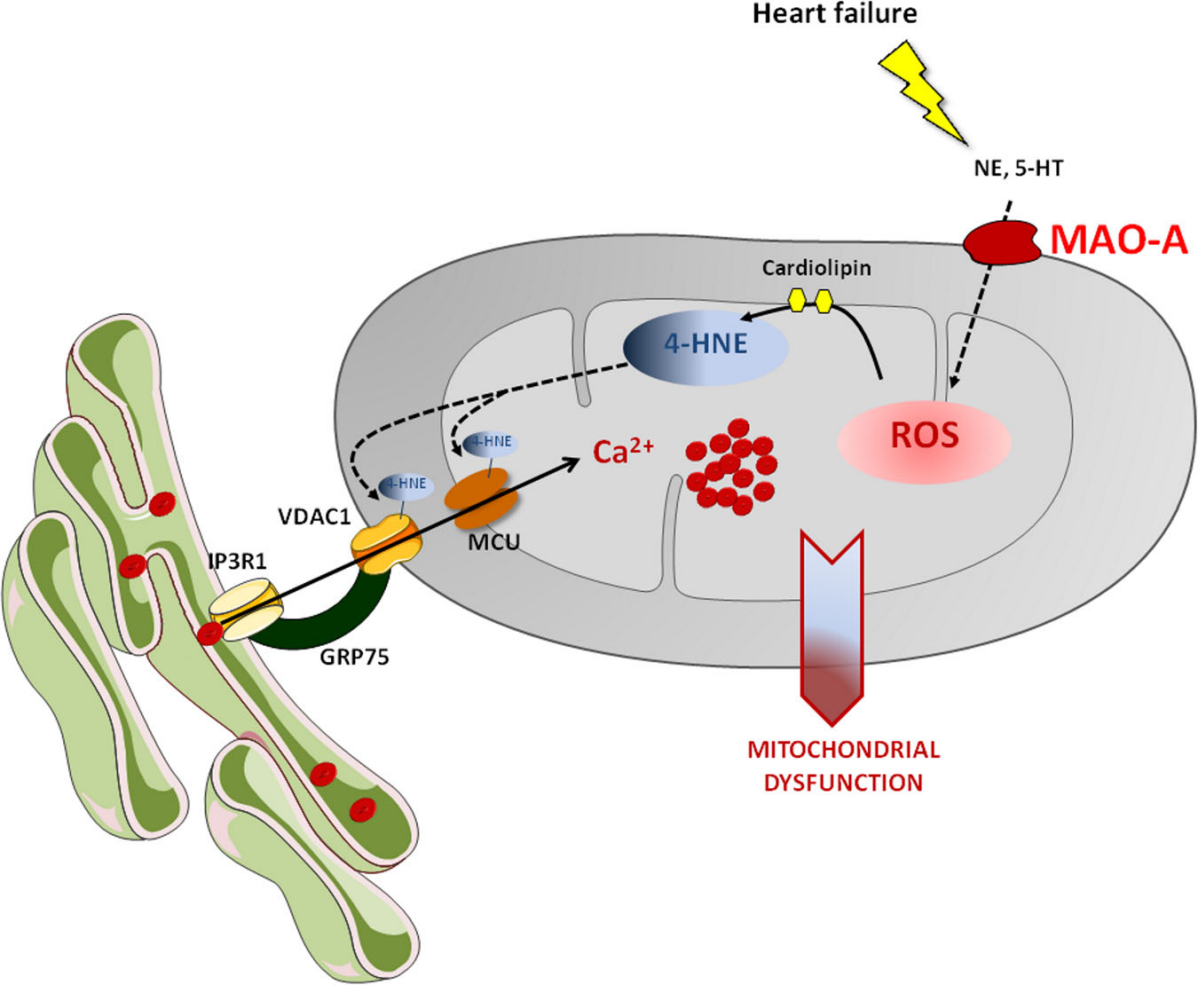
MAO-A at the crossroad of mitochondrial Ca^2+^ and ROS. During heart failure, an increase in MAO-A substrates together with enhanced MAO-A expression leads to the accumulation of H_2_O_2_ into the mitochondria. Subsequently, ROS-mediated peroxidation of cardiolipin enhances the production of 4-HNE which binds to VDAC (voltage-dependent anion channel) and MCU (mitochondrial Ca^2+^ uniporter). A resulting increase in Ca^2+^ uptake leads to Ca^2+^ overload and mitochondrial dysfunction with ATP depletion and loss of mitochondrial membrane potential.

In conclusion, we provide a new comprehension of the molecular steps that go from the activation of MAO-A to the disruption of mitochondrial function and HF (Fig. 1)^11^. MAO-A inhibition is protective in the setting of different cardiac stresses such as pressure overload HF, diabetic cardiomyopathy and now chronic ischemia, indicating its central role in deleterious ROS production and mitochondrial dysfunction^8,11^. In addition, MAO-A seems to regulate all the different processes of mitochondrial quality control such as mitochondrial biogenesis through PGC1α, mitochondrial function and mitophagy^8,10^. In a therapeutic point of view, we found that the administration of moclobemide, a MAO-A selective and reversible inhibitor, which is the active compound of moclamine drug, an antidepressant used in Europe, prevented cardiac dysfunction, lung congestion and ventricular remodeling in mice with chronic cardiac ischemia. It would be interesting to consider the possibility of repurposing this drug for heart therapy in the future.
